# Breaching the Bacterial Envelope: The Pivotal Role of Perforin-2 (MPEG1) Within Phagocytes

**DOI:** 10.3389/fimmu.2021.597951

**Published:** 2021-02-22

**Authors:** Leidy C. Merselis, Zachary P. Rivas, George P. Munson

**Affiliations:** Department of Microbiology and Immunology, Leonard M. Miller School of Medicine, University of Miami, Miami, FL, United States

**Keywords:** pore-forming protein, MACPF/CDC family proteins, innate immune effector, phagosome, phagocyte, perforin-2/MPEG1, macrophage

## Abstract

The membrane attack complex (MAC) of the complement system and Perforin-1 are well characterized innate immune effectors. MAC is composed of C9 and other complement proteins that target the envelope of gram-negative bacteria. Perforin-1 is deployed when killer lymphocytes degranulate to destroy virally infected or cancerous cells. These molecules polymerize with MAC-perforin/cholesterol-dependent cytolysin (MACPF/CDC) domains of each monomer deploying amphipathic β-strands to form pores through target lipid bilayers. In this review we discuss one of the most recently discovered members of this family; Perforin-2, the product of the *Mpeg1* gene. Since their initial description more than 100 years ago, innumerable studies have made macrophages and other phagocytes some of the best understood cells of the immune system. Yet remarkably it was only recently revealed that Perforin-2 underpins a pivotal function of phagocytes; the destruction of phagocytosed microbes. Several studies have established that phagocytosed bacteria persist and in some cases flourish within phagocytes that lack Perforin-2. When challenged with either gram-negative or gram-positive pathogens *Mpeg1* knockout mice succumb to infectious doses that the majority of wild-type mice survive. As expected by their immunocompromised phenotype, bacterial pathogens replicate and disseminate to deeper tissues of *Mpeg1* knockout mice. Thus, this evolutionarily ancient gene endows phagocytes with potent bactericidal capability across taxa spanning sponges to humans. The recently elucidated structures of mammalian Perforin-2 reveal it to be a homopolymer that depends upon low pH, such as within phagosomes, to transition to its membrane-spanning pore conformation. Clinical manifestations of *Mpeg1* missense mutations further highlight the pivotal role of Perforin-2 within phagocytes. Controversies and gaps within the field of Perforin-2 research are also discussed as well as animal models that may be used to resolve the outstanding issues. Our review concludes with a discussion of bacterial counter measures against Perforin-2.

## Introduction

Perforin-2 is a member of the Membrane Attack Complex, Perforin/Cholesterol-Dependent Cytolysin (MACPF/CDC) superfamily of proteins (1). Most, but not all, members of this family are pore-forming proteins (2–6). This includes lytic bacterial toxins such as pneumolysin and perfringolysin O, as well as innate immune effectors such as complement protein C9 and Perforin-1. In the blood C9, together with other complement proteins, forms the membrane attack complex (MAC) to perforate the envelope of gram-negative bacteria (7, 8). Perforin-1 is deployed when killer lymphocytes degranulate to destroy virally infected or cancerous cells (9, 10). These molecules polymerize into rings with inner diameters of 120–300 Å (7, 10–12). Pores are formed when each MACPF deploys four amphipathic β-strands through lipid bilayers to form the β-barrel of the pore.

The gene encoding Perforin-2, *Mpeg1*, was first described in 1995 as a highly expressed gene within macrophages (13). After noting the presence of a MACPF domain the authors proposed that Perforin-2 was likely another pore-forming protein. However, more than a decade would lapse before functional, mechanistic, and structural research would begin in earnest. It is now clear that *Mpeg1* is a primordial gene present in taxa spanning sponges to humans. Its domain organization has remained little changed by evolution except in cases of gene duplication. In such cases the paralog may diverge from *Mpeg1*. Indeed analyses across taxa and gene families suggest *Mpeg1* is the ancestor of Perforin-1 and MACPF-containing complement proteins (14). In this review we critically evaluate recent progress in the nascent but growing field of Perforin-2 research with an emphasis on its expression and function within phagocytic cells.

## Perforin-2 Structure and Cellular Location

**Figure 1:**
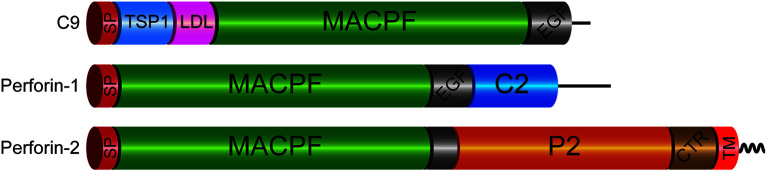
Domain Organization of Mammalian C9 and Perforins. Each of the immune effectors contains a signal peptide (SP), membrane attack complex perforin (MACPF), and epidermal growth factor-like (EGF) domains. However, the latter is truncated in Perforin-2. The P2 domain is unique to Perforin-2 and has been evolutionarily conserved across taxa. Recent structural and functional studies suggest the P2 domain initiates contact with target membranes. However, this does not preclude other putative functions such oligomerization and/or ring stabilization. Another distinctive feature of Perforin-2 is a transmembrane domain (TM) near its carboxy terminus. Although Perforin-2 is initially a Type I transmembrane protein, it is likely cleaved from its TM domain as it is delivered to phagosomes to facilitate oligomerization and pore formation. The TM domain is followed by a short cytosolic tail that is involved in the intracellular trafficking of Perforin-2 to phagosomes. TSP1, thrombospondin type-1 repeat; LDL, low-density lipoprotein receptor class A repeat; C2, calcium-dependent phospholipid binding domain; CTR, carboxy-terminal region.

Unlike soluble Perforin-1 and C9, Perforin-2 is a type I transmembrane protein (**Figure 1**). In this orientation the MACPF of Perforin-2 resides within the lumen of vesicular structures. As determined by subcellular fractionation of human macrophages, endogenous Perforin-2 colocalizes with markers of the ER, Golgi, endosomes, and phagosomes (15). A proteomic study identified Perforin-2 (referred to as MPS1 in that study) in the phagolysosome compartment of activated murine macrophages (16). Another analyzed bone marrow derived dendritic cells and reported that Perforin-2 co-resides with subunits of the phagocytic NAPDH oxidase and other antimicrobial effectors of endo/phagosomes (17). Moreover, LPS stimulation increased the abundance of Perforin-2 within those vesicles. A third proteomic study found Perforin-2 within macrophage endo/phagosomes following phagocytosis of latex beads (18). Consistent with the studies above, a Perforin-2-RFP fusion protein was shown to co-localize with phagocytosed bacteria (15). This is unlikely to be an artefact because the fusion protein was also shown to be bactericidal against phagocytosed bacteria. In aggregate these studies provide compelling evidence that Perforin-2 is trafficked to endo/phagosomes.

**Figure 2:**
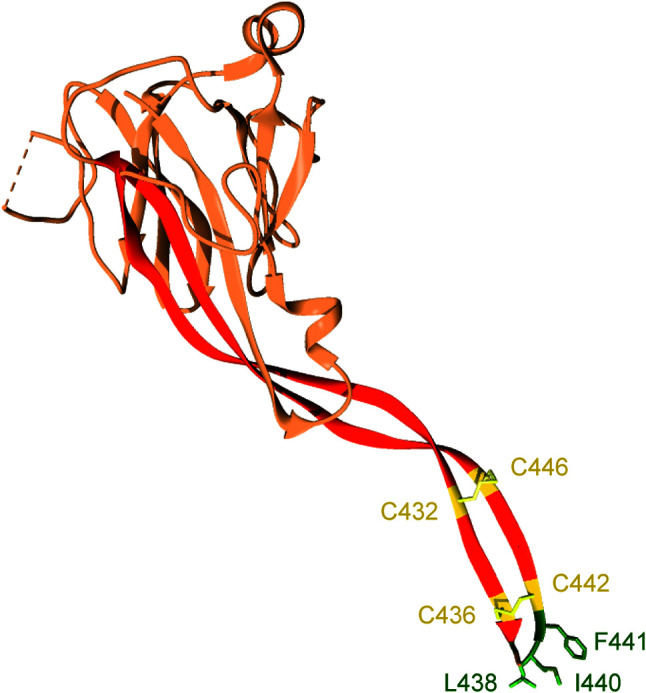
The P2 domain of murine Perforin-2. The most prominent feature within the P2 domain is an extended β-hairpin; highlighted in dark orange. Two disulfide bonds stiffen the β-hairpin. Hydrophobic residues at the β-hairpin’s tip are shown in dark green. These residues likely orient Perforin-2 on phagocytosed bacteria by initiating contact with target lipid bilayers. Numbering is relative to UniProt accession number A1L314. This graphic was rendered with UCSF Chimera from PDB file 6SB1 (http://www.rcsb.org/structure/6SB1) (20).

The transmembrane domain of Perforin-2 is followed by a cytosolic tail; typically, of less than 40 residues. As discussed below this short cytosolic tail is involved in the intracellular trafficking of Perforin-2 (19). In addition to the loss of the transmembrane domain, Perforin-1 and C9 have also lost the P2 domain. This latter domain is conserved across taxa and to date has not been found in any gene other than *Mpeg1*. The function of the novel P2 domain was only recently investigated through structural and mechanistic studies (20, 21). Its most prominent feature is an extended β-hairpin—stiffened by inter-strand disulfide bonds—that culminates in a hydrophobic tip (**Figure 2**) (20). The extended β-hairpin is likely involved in the initial interactions with membranes as determined by liposome binding studies with the isolated P2 domain (20). As expected, liposome binding was abolished by deletion of the β-hairpin (20). Consistent with the composition of bacterial membranes, the P2 domain was also found to preferentially bind liposomes containing negatively charged lipids (20). Although more work is required these studies suggest that the P2 domain mediates Perforin-2’s initial interactions with target membranes. Moreover, its extended β-hairpin may be functionally analogous to domain 4 of cholesterol-dependent cytolysins. Domain 4 contains the cholesterol binding motif as well as a signature undecapeptide at the tip of the domain that anchors the cytolysins to the target membrane (22, 23).

**Figure 3:**
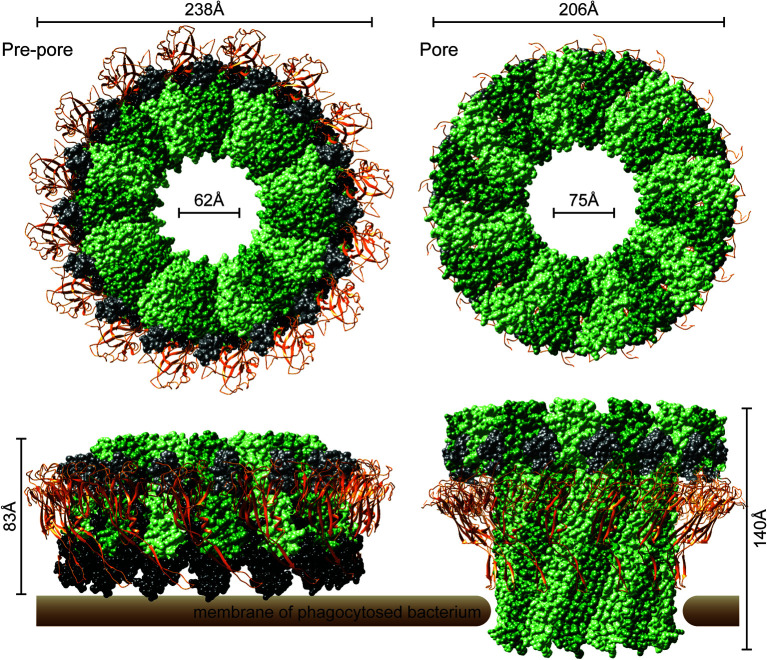
The pre-pore and acid-dependent pore of murine Perforin-2 from top and side views. Each polymer is composed of 16 subunits. MACPF domains line the interior of each polymer and are depicted in alternating shades of green. P2 domains are depicted as orange ribbons that encircle the exterior of each polymer. A truncated EGF domain, light gray, links the MACPF and P2 domains. The carboxy-terminal region is shown in black. This region is visible in the pre-pore but was not resolved in the pore. Images were rendered with UCSF Chimera from PDB files 6SB3 (http://www.rcsb.org/structure/6SB3) and 6SB5 (http://www.rcsb.org/structure/6SB5) (20).

**Figure 4:**
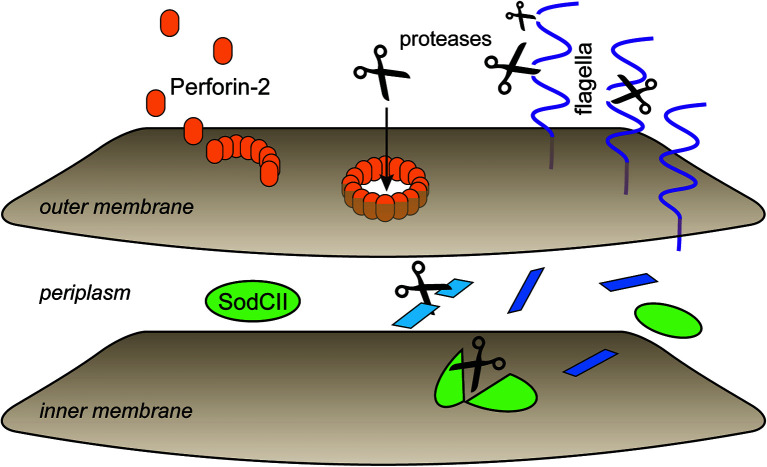
Perforin-2 facilitates protease entry to the periplasmic space. Shortly after microbe phagocytosis Perforin-2 is delivered to the phagosome and deposits on the bacterial envelope. As the phagosome acidifies pre-pores transition to pores that breach the outer membrane. This allows proteases to enter the periplasmic space and begin the digestion of periplasmic and inner membrane proteins. In the absence of Perforin-2 phagosomal proteases are restricted to digestion of outer membrane proteins such as flagellin. This graphic is an adaptation from a previously published version (26).

In the recently determined structures of polymerized human and mouse Perforin-2 the P2 and MACPF domains form the exterior and interior rings of the polymer respectively (**Figure 3**) (20, 21). On average these rings are composed of 16 monomers with a height of 83 Å in the pre-pore conformation (20, 21). The pre-pore to pore transition is accompanied by a dramatic 170% increase in height as each monomer deploys its four amphipathic β-strands (**Figure 3**) (20). These β-strands align with each other and those of neighboring subunits to form the barrel of the pore. Acidic pH drives the pre-pore to pore transition (20, 21). This trigger is biologically relevant because it has long been established that phagosomes rapidly acidify and Perforin-2 has been shown to colocalize with phagocytosed bacteria such as *Salmonella enterica* serovar Typhimurium; hereafter *S*. Typhimurium (15, 24, 25). A separate study—graphically summarized in **Figure 4**—found that periplasmic proteins of *S*. Typhimurium were efficiently degraded within the phagosomes of wild-type macrophages and neutrophils (26). In contrast, the degradation of periplasmic proteins was delayed within the phagosomes of *Mpeg1*−/− phagocytes. This was not due to differences in phagosomal proteases because a surface marker (flagellin) was efficiently degraded in both wild-type and Perforin-2 deficient phagocytes. Thus, the *in situ* observations are consistent with Perforin-2 pores breaching the outer membrane of *S*. Typhimurium allowing the passage of phagosomal hydrolases to the periplasmic space.

## Perforin-2 in Non-Mammalian Species

### *Mpeg1* Expression in Invertebrates and Bony Fish

As in other animals the innate immune responses of invertebrates and bony fish provide protection against pathogenic threats. Some of those responses involve changes within the transcriptome and pathogen or PAMP induced expression can be indicative of a gene’s immunological role. *Mpeg1* expression is upregulated in the sponge *Suberites domuncula* following LPS challenge relative to untreated animals (27). Similarly, LPS has been shown to induce the expression of *Mpeg1* in the stony coral *Pocillopora damicornis* (28). *Mpeg1* mRNA was significantly upregulated in the brain, head kidney, heart, liver, intestine, and spleen of the starry flounder *Platichthys stellatus* following infection with *Streptococcus parauberis* (29). Relative to untreated controls the expression of *Mpeg1* is significantly increased when the Mediterranean mussel *Mytilus galloprovincialis* is exposed to heat-killed *Vibrio anguillarum* (30). Likewise infection of the disk abalone *Haliotis discus discus* with either gram-negative *Vibrio parahaemolyticus* or gram-positive *Listeria monocytogenes* induces the expression of *Mpeg1* (31). Transcriptome analysis of the larvae of the eastern oyster *Crassostrea virginica* revealed that exposure to either the gram-negative *Phaeobacter inhibens* or gram-positive *Bacillus pumilus* induces expression of *Mpeg1* (32). Similar results were observed when another species of mollusk, *Haliotis midae*, was challenged with live *V. anguillarum* (33). In the latter study increased transcription of *Mpeg1* corresponded with elevated levels of Perforin-2. Of the studies discussed directly above the latter was the only one to evaluate expression at the protein level.

The genome of the orange spotted grouper, *Epinephelus coioides*, harbors two copies of *Mpeg1*. Relative to other organs both are constitutively expressed at high levels in the spleen and head kidney (34). Hematopoiesis occurs in the latter organ and it is analogous to mammalian bone marrow (35). Both copies were significantly upregulated in the spleen and gills following challenge with *Cryptocaryon irritans*, a protozoan parasite of significant concern to the aquaculture industry (34, 36). These results were subsequently confirmed by immunofluorescence with polyclonal antibodies that recognize both isoforms of *E*. *coiodes* Perforin-2 (37). In aggregate studies across species of invertebrates and bony fish have shown that infection and/or PAMPs induce the expression of *Mpeg1*.

Although infection or PAMP induced expression of *Mpeg1* suggests Perforin-2 plays a role in host defense, *Mpeg1* downregulation following bacterial infection may be indicative of bacterial countermeasures. For example, in *S. domuncula* expression of *Mpeg1* is dampened by the bacterial sponge pathogen *Pseudoalteromonas* sp., but not the commensal bacterium *Endozoicomonas* sp.; species were indeterminate (38). This differential effect is suggestive of a pathogenic countermeasure deployed to defeat Perforin-2. Reduced *Mpeg1* expression has also been documented in the corals *Acropora cerviconis* and *Acropora palmata* when they present with white band disease (28, 39). Although the etiological agent of white band disease is currently unknown, it has been suggested to be bacterial (40). Although the number of studies is few and the data preliminary, they raise the possibility that certain species of pathogenic bacteria may suppress the expression of *Mpeg1* to promote colonization of invertebrates. Whether this is through stealth strategies, such as LPS modifications, or active counter measures, such as effector proteins and toxins, remains to be elucidated.

### Analyses of Recombinant Perforin-2 From Invertebrates and Bony Fish

Seminal studies have reconstituted the activity of the complement membrane attack complex and Perforin-1 *in vitro* (7, 12, 41, 42). In recent years researchers have attempted to extend such analyses to Perforin-2. Although the P2 domain of Perforin-2 is evolutionarily conserved from sponges to humans, three studies dispensed with it to evaluate the activity of the MACPF domain from abalone, *H. discus discus*, oyster *Crassostrea gigas*, or flounder *Platichthys stellatus* (29, 31, 43). These studies reported at least some activity against gram-negative (*Edwardsiella piscicida, Escherichia coli*, *Vibrio anguillarum, Vibrio campbelli, Vibrio harveyi, Vibrio ordalii*, *Vibrio tapetis*, and *Vibrio alginolyticus*) and gram-positive (*Streptococcus iniae*, *Streptococcus parauberis*, *Staphylococcus aureus*, *Bacillus thuringiensis*, and *Bacillus subtilis*) bacteria. Others have examined the antibacterial effects of mostly full-length Perforin-2; minus signal peptides, transmembrane domains and carboxy terminal residues (27, 34). Recombinant Perforin-2 from sponge, *S. domuncula*, was reported to have a negative effect upon *E. coli* K-12 and B; but not gram-positive *Staphylococcus aureus* (27). In contrast, Perforin-2a from orange spotted grouper, *E. coiodes*, was reported to inhibit the growth of both gram-negative and gram-positive bacteria (34). *E. coiodes* Perforin-2b was found to be active against only gram-positive bacteria (34). This difference is surprising because the two proteins have 90% overall identity. Nevertheless, differences were also observed when the two proteins were tested against parasitic *C. irritans*. Perforin-2b has no activity against *C. irritans*. In contrast Perforin-2a inhibited motility and caused rounding of theronts, the free-swimming infective form of the parasite. This rounding led the authors to speculate that Perforin-2a decreases *C. irritans* infectivity although this was not directly tested (34).

Although each of the above studies claim that recombinant Perforin-2, or its MACPF, has antimicrobial activity, each has methodological and analytical weaknesses. For example, each recombinant protein was expressed in *E. coli* and thus would lack their usual post-translational modifications. The MACPFs of complement protein C9 and Perforin-1 are known to be glycosylated (10, 44–49). Likewise, the MACPFs of Perforin-2 from sponges to humans are predicted to be N-glycosylated at two or more Asn residues (http://www.cbs.dtu.dk/services/NetNGlyc/). In the case of murine and human Perforin-2 these modifications have been confirmed and may play critical roles in folding and/or pore formation (20, 21, 50, 51). It is also important to point out that none of the four studies reported bacterial killing. Rather, three simply measured the optical absorbance of bacterial cultures (31, 34, 43). The fourth did quantify CFUs but only after overnight incubation with the protein (27). Thus, in all four cases it is unknown if the reported effects are due to bacterial death or growth inhibition.

Although it is impossible to discern the mechanism(s) of the reported effects, we can deduce that pore formation was probably not involved when only the MACPF domain of Perforin-2 was used (31, 43). Structural studies have shown that the P2 domain is likely intrinsic to polymerization and pore formation as it forms the outer ring of both pre-pores and pores with extensive surface area contacts between adjacent P2 domains and MACPFs (**Figure 3**) (20, 21). Phospholipid and liposome binding studies have also revealed that the P2 domain (**Figure 2**) is likely required for initiating interactions with the bacterial envelope (20, 21). The reported activity against gram-positive bacteria is also unexpected because they are surrounded by thick layers of peptidoglycan (27, 31, 34, 43). Although Perforin-2 does kill phagocytosed gram-positive bacteria, this likely requires the assistance of phagosomal hydrolases to degrade the peptidoglycan barrier (15, 52–54).

## Animal Models for Perforin-2 Research

### Zebrafish

**Figure 5:**
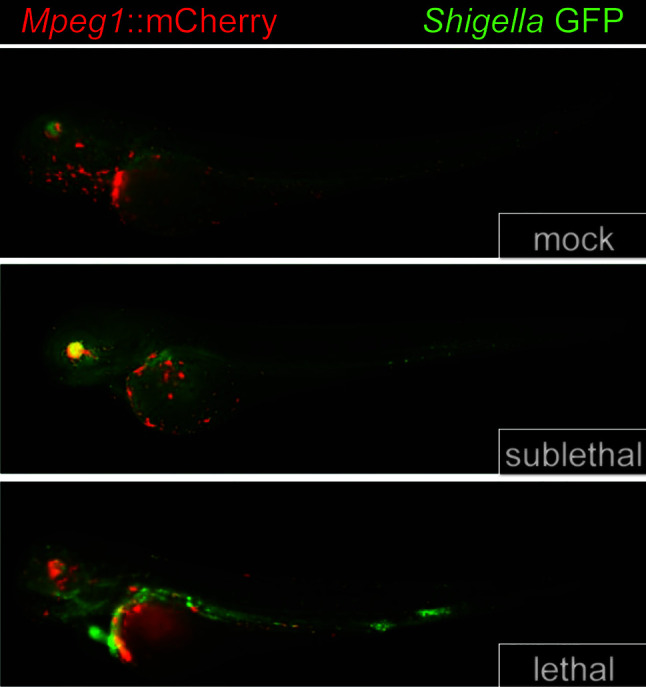
*Mpeg1::mCherry* reporters facilitate the tracking of macrophages in larval zebrafish. Because the expression of Perforin-2 is largely restricted to macrophages in zebrafish, the insertion of *mCherry* into the *Mpeg1* locus allows localization of macrophages in both fixed and live animals. The transgenic animals above were infected with sublethal and lethal doses of *Shigella flexneri* and fixed 24 h post infection. The above images have been previously published (58) and are a composite of the red and green fluorescent channels. This figure was adapted from source images posted at https://doi.org/10.1371/journal.ppat.1003588.g002 under the CC BY 4.0 license.

Zebrafish have three *Mpeg1* paralogs: *Mpeg1, Mpeg1.2, and Mpeg1.3* (55, 56). Both *Mpeg1* and *Mpeg1.2* are expressed in macrophages (57). In contrast, transcriptomic studies of both larval and adult zebrafish have determined that *Mpeg1.3* is a silent gene (57). *Mpeg1* is under the control of the spi/pu.1 transcription factor which also regulates the expression of genes associated with myeloid differentiation (56). Thus, fluorescent reporters such as mCherry can be used to track macrophages *in situ* when the reporter is expressed from the *Mpeg1* promoter (56) (**Figure 5**). *Mpeg1.2* expression is upregulated during infection with mycobacteria, gram-negative and gram-positive bacteria (57). Curiously, the same bacterial infections inhibit *Mpeg1* expression (57). This expression pattern is unlikely the result of bacterial effectors or toxins, as *Mpeg1* downregulation was also observed when zebrafish were challenged with heat killed or avirulent bacteria.

Although *Mpeg1* and *Mpeg1.2* are inversely expressed during bacterial infections, both genes produce antibacterial responses (57). Zebrafish embryos treated with *Mpeg1* specific morpholinos have increased intracellular loads of *S.* Typhimurium and *Mycobacterium marinum* relative to control morpholinos. Likewise embryos treated with *Mpeg1.2* morpholinos succumb to bacterial infection significantly earlier than *Mpeg1* knockdowns and controls (57). It was also observed that *Mpeg1.2* has a greater antibacterial contribution than *Mpeg1* which is consistent with their expression patterns during infection (57). Curiously, *Mpeg1* knockdowns survive infections longer than control embryos despite greater bacterial burden. It has been proposed that *Mpeg1* is a broad regulator of innate immune responses that promotes survival by diminishing lethal inflammatory responses while *Mpeg1.2* is directly responsible for the bactericidal effects (57). This is surprising given that the two proteins share a common domain architecture and are 80%–90% identical. Additional experimentation is required to clarify the roles of *Mpeg1* and *Mpeg1.2* during infection.

### Transgenic Mice

Eckhard R. Podack (1943–2015), in a remarkably productive collaboration with his trainee Dr. Ryan M. McCormack (59), was the first to demonstrate that mammalian Perforin-2 plays a pivotal role in the elimination of intracellular bacteria including MRSA, *Mycobacterium smegmatis*, and *S*. Typhimurium (15, 53). The Podack laboratory was also the first to report colocalization of Perforin-2 with phagocytosed bacteria (15), and interferon induced expression of *Mpeg1* in keratinocytes, fibroblast and a plethora of other cell types (15). Prof. Podack was also the first to demonstrate that bacterial challenge elicits the expression of *Mpeg1* in murine embryonic fibroblasts (53). Prof. Podack also coined the moniker “Perforin-2” and vigorously advocated for its usage because he understood that Perforin-2 is more descriptive of the protein’s function than macrophage-expressed gene 1 protein (MPEG1) (60, 61). When coupled with his lifelong interest in MACPFs of the immune system (62–71), these and other foundational contributions to the field motivated Prof. Podack to commission the development of *Mpeg1* knockout mice at the University of Miami Miller School of Medicine, USA. When raised under specific pathogen-free conditions these knockout mice develop normally and are phenotypically indistinguishable from their wild-type counterparts. However, in another seminal publication—and his last as sole corresponding author — Prof. Podack reported that Perforin-2 deficient mice succumb to low dose bacterial infections that most wild-type mice survive (15). As described below these results are not limited to a particular route of infection or pathogen.

#### Orogastric Inoculation of Enteric, Gram-Negative Pathogens

**Figure 6:**
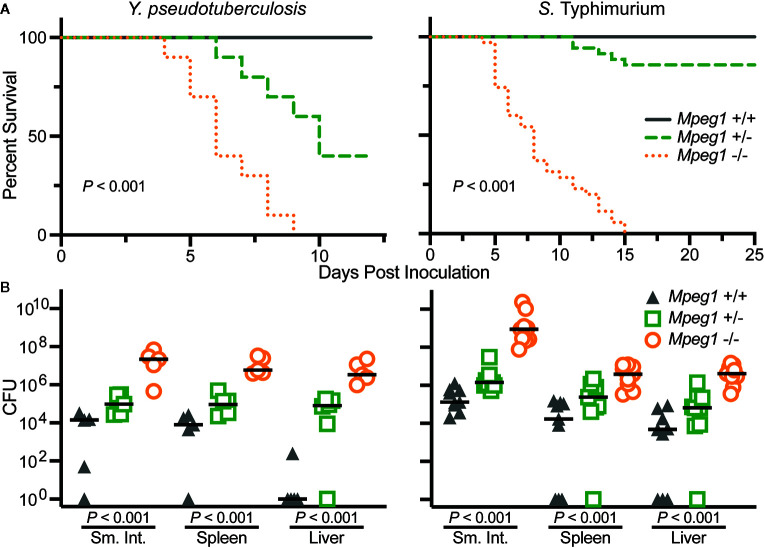
Peforin-2 deficient mice are immunocompromised**. (A)** Survival curves of wild-type, *Mpeg1* +/−, and −/− C57Bl/6 x 129X1/SvJ mice after orogastric inoculation with 10^6^ CFU *Y. pseudotuberculosis* or 10^5^ CFU *S*. Typhimurium. *P* values determined by Log-rank (Mantel-Cox) test. **(B)** Organ loads of wild-type, *Mpeg1* +/−, and −/− C57Bl/6 x 129X1/SvJ mice after orogastric inoculation with 10^6^ CFU *Y. pseudotuberculosis* or 10^5^ CFU *S*. Typhimurium. The former group of animals were sacrificed 10 days post inoculation while the latter were sacrificed 5 days post inoculation. Horizontal bars denote the medians. *P* values were determined by non-parametric Kruskal-Wallis test. This figure was adapted from previously published work (15, 19) from source data posted at https://doi.org/10.35092/yhjc.12584993.v1 under the CC BY 4.0 license. Sm. Int., small intestine.

Wild-type, *Mpeg1* +/− and −/− mice have been orogastrically challenged with the enteric pathogens *Yersinia pseudotuberculosis* and *S.* Typhimurium (15, 19). In both cases all *Mpeg1* −/− mice succumbed to infection within 15 days of inoculation (**Figure 6**). In contrast all wild-type mice survived sublethal challenges. Heterozygous mice revealed a gene dosage effect with survival profiles between wild-type and *Mpeg1* −/− mice. Perforin-2 deficiency also correlated with significantly higher loads of the pathogens in the intestines and dissemination to deeper tissues such as spleens and livers (**Figure 6**) (15, 19). Thus, two independent studies have demonstrated that Perforin-2 deficient mice are immunocompromised and unable to control infections that their wild-type cohorts survive (15, 19). These findings are further supported by *in vitro* studies which have demonstrated that Perforin-2 deficient phagocytes and fibroblasts are less efficient killers of intracellular bacteria than wild-type cells (15, 26, 52, 53, 72).

#### *Chlamydia* Intravaginal Infection

Because phagocytes limit chlamydiae infections, investigators evaluated the role of Perforin-2 in an intravaginal infection model with *Chlamydia muridarum*; a gram-negative, obligate intracellular pathogen (73, 74). In addition, cell based assays had previously established that Perforin-2 limits the growth of chlamydiae in macrophages (72). In the intravaginal model *Mpeg1* −/− mice exhibited significantly greater weight loss than wild-type controls and displayed other signs of morbidity such as ruffled fur (74). Surprisingly, there was no difference in the time to resolution — as determined by shedding of inclusion forming units, a quantitative indicator of infectivity and/or transmissibility—between the two groups. However, the researchers also observed that Perforin-2 deficient mice shed less inclusion forming units; especially, at mid-time points of the infection. To explain this conundrum the researchers speculate that *C. muridarum* may more easily ascend the genital tract and disseminate in Perforin-2 deficient than wild-type mice (74). However the experiment was not designed to test that hypothesis. Therefore, it will be necessary to revisit this model and monitor dissemination to peripheral sites to determine whether or not *C. muridarum* does disseminate in Perforin-2 deficient mice.

#### Epicutaneous Infection With MRSA

*S. aureus* is a gram-positive bacterium often present on human skin as a part of the dermal microbiome. To evaluate the role of Perforin-2 at the dermal barrier investigators shaved mice then used tape to disrupt the epidermal barrier prior to administering methicillin resistant *S. aureus* (MRSA) (15). As with other infection modalities the vast majority of infected *Mpeg1* −/− mice perished; although, the time to death was significantly delayed compared to the orogastric models discussed above. In contrast, ca. 80%–100% of the heterozygous and wild-type mice survived MRSA challenge. In another experiment the skin of *Mpeg1* −/− mice contained 3 logs more MRSA than that of wild-type or heterozygous mice (15). As expected, Perforin-2 deficiency was accompanied by bacterial dissemination to the blood, spleen, and kidneys. MRSA may manipulate the transcriptome of host cells to promote its own survival because the pathogen was shown to decrease the expression of *Mpeg1* in human skin cells (75). However, pretreating human skin cells with the commensal bacterium *S. epidermidis* prior to MRSA infection led to increased *Mpeg1* expression and enhanced killing of intracellular bacteria.

#### Intravenous Delivery of *Listeria*

Perforin-2 has also been shown to aid in defense against another gram-positive bacterium, *Listeria monocytogenes.* Perforin-2 deficient mice infected intravenously with *L. monocytogenes* have significantly greater loads of the pathogen in their spleens and livers than wild-type mice (52). In a pregnancy model of infection Perforin-2 deficient mice had significantly higher loads of *L. monocytogenes* in both the placenta and fetuses (76). In these models injected bacteria are phagocytosed and killed by splenic and liver macrophages (77). But some phagocytosed bacteria escape to the cytosol where they replicate and disseminate to other cells *via* actin polymerization (78). To escape the phagosomal vacuole *L. monocytogenes* deploys its own pore-forming protein, the cholesterol-dependent cytolysin listeriolysin O (79). Timing is likely critical: *L. monocytogenes* must escape the vacuole before Perforin-2 delivers its lethal blow. Consistent with this hypothesis significantly more bacteria escape to the cytosol of *Mpeg1* −/− than wild-type macrophages in cell based assays (52).

In the above experiments the authors also observed that phagocytosed *L. monocytogenes* were more likely to reside within acidic vacuoles of Perforin-2 deficient macrophages than wild-type macrophages (52). To explain this phenomenon the authors proposed that Perforin-2 limits vacuole acidification. This is controversial as to date there is no mechanistic evidence to support that hypothesis. Rather, the recent discovery that acid drives the Perforin-2 pre-pore to pore transition suggests an alternative hypothesis (20, 21). In our reinterpretation of the available data we propose that fewer *L. monocytogenes* reside within acidic vacuoles because acid activates Perforin-2 which then facilitates the destruction of vacuolar bacteria. In the absence of Perforin-2 bacteria are simply able to persist longer within acidic vacuoles. We also note that another study found that the acidification of *Salmonella* containing vacuoles was equivalent between wild-type and Perforin-2 deficient macrophages; see Bai et al., 2018, Supplementary Materials (26).

#### Contradictory Signals: Perforin-2 and Type I IFN Signaling

Although *Mpeg1* is an IFN stimulated gene (15, 53), it has also been reported that Perforin-2 is required for Type I IFN signaling by forming complexes with IFN receptors IFNAR1 and IFNAR2 (80). The impetus for that study was the observation that *Mpeg1* deficient mice are resistant to LPS induced septic shock; a model in which Type I IFNs play a central role in driving the cytokine storm. However other studies found that *Mpeg1* deficient mice, on either C57BL/6 or 129X1/SvJ backgrounds, are not more resistant to LPS induced septic shock than wild-type animals (81). As validation of the latter study’s experimental design and outcome, both wild-type and knockout mice on the 129X1/SvJ genetic background were more resistant to LPS than C57BL/6 mice. This effect is consistent with previous studies and is due to the fact that 129X1/SvJ mice lack caspase-11 (82, 83).

**Figure 7:**
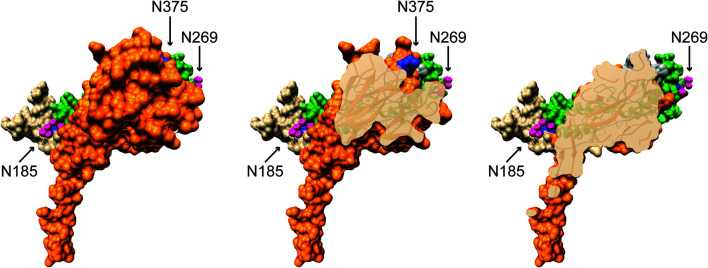
Glycosylated Asn185 and Asn269 are located on opposite faces of the MACPF domain. A monomer of murine Perforin-2 shown in (Left) full view and (Middle & Right) progressive cross-sections into the molecule. The MACPF and P2 domains are shaded green and orange respectively. The truncated EGF domain, which connects the MACPF and P2 domains, is shaded grey and is partially visible in the upper right of the far right image. The extended carboxy terminal region is shown in tan. Asn185 and 269 are conserved and glycosylated in both murine and human Perforin-2 (20, 21). Their glycans are shown as pink spheres. In contrast to the MACPFs, the P2 domains of both species are devoid of glycosylation. This includes the absence of glycosylation of Asn375; shaded deep blue above and visible in the middle image. Images were rendered with UCSF Chimera from PDB file 6SB3 (http://www.rcsb.org/structure/6SB3) (20). Residue numbering is relative to UniProt entry A1L314 (https://www.uniprot.org/uniprot/A1L314).

Further contradicting Perforin-2’s role in Type I IFN signaling, an RNAseq study found that Type I IFN signaling is functional in *Mpeg1* −/− murine phagocytes stimulated with IFN-β (84). It is also difficult to reconcile the proposed complexes with the structures of Perforin-2 and IFNARs (20, 21, 80, 85). For example, it was reported that binding to and signaling through IFNAR1 requires glycosylation of Perforin-2 residues Asn185 and Asn269; numbering relative to UniProt entry A1L314 (https://www.uniprot.org/uniprot/A1L314) (80). Although the structures of both mouse and human Perforin-2 confirm that both residues are glycosylated, Asn185 and Asn269 reside on opposite faces of the MACPF domain (**Figure 7**) (20, 21). Not surprisingly, docking simulations with the known structures of IFNAR1 and Perforin-2 monomers found no plausible pathway for simultaneous binding to Asn185 and Asn269 by IFNAR1 (86). Thus, it is not clear how IFNAR1 is able to contact both as has been proposed (80). Likewise, it has been reported that glycosylation of Asn375 in the P2 domain is essential for interactions with IFNAR2 (80). This residue is visible in all published structures of murine and human Perforin-2 but unlike Asn185 and Asn269, Asn375 is not glycosylated (**Figure 7**, middle image) (20, 21). Although it is possible that the glycosylation pattern of Perforin-2 differs between expression systems and cell lines, bioinformatics suggests otherwise. Like other servers, NetNGlyc identifies Asn-X-Ser/Thr sequons (http://www.cbs.dtu.dk/services/NetNGlyc/). However, its neural network also evaluates the surrounding sequences to predict the probability of glycosylation. In agreement with the structural studies (20, 21). NetNGlyc predicts glycosylation of Asn185 and Asn269 but not Asn375. In summary, the reported requirement for Perforin-2 in Type I IFN signaling and proposed mechanism (80) are challenged by transcriptomics, LPS induced sterile septic shock, and molecular analyses. Clearly additional studies are required to resolve these contradictions.

#### *PfpL*, A Paralog of Murine *Mpeg1*

Unlike humans, mice have a paralog of *Mpeg1* named Pore-Forming Protein Like (*PfpL*, UniProt entry Q5RKV8). Over their entire length PfpL and murine Perforin-2 are 65% identical. This suggest that PfpL could also function as an immune effector. However, to date there have been no functional studies of PfpL and expression of the *PfpL* transcript appears to be more limited than that of *Mpeg1* as determined by the murine gene expression database, GXD (87). Unlike *Mpeg1*, *PfpL* expression has only been observed in the context of murine development and in the adult mouse liver. Particularly high expression was also observed in a subset of trophoblast giant cells and the parietal yolk sac (88). Further experiments are needed to determine whether or not PfpL is a functional immune effector. In addition, greater clarity is required regarding the timing and location of its expression. However, the restricted expression of *PfpL* and the clear immunocompromised phenotype of *Mpeg1* knockout mice suggest *PfpL* is at best a minor player in host defense under most circumstances.

## Clinical Impacts of *Mpeg1* Missense and Nonsense Mutations

**Figure 8:**
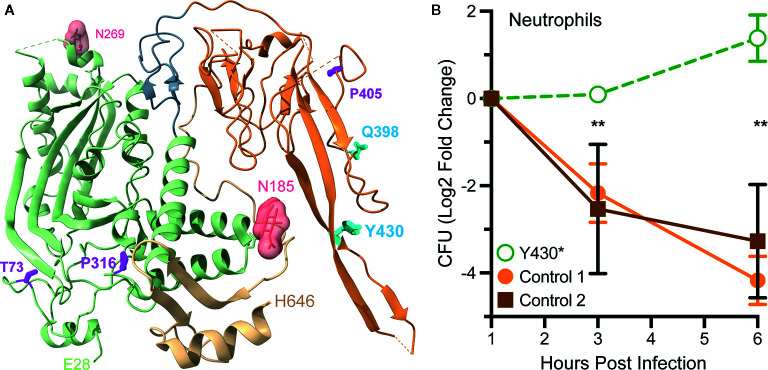
Perforin-2 haploinsufficiency results in the reduced ability of human phagocytes to kill intracellular bacteria. **(A)** A monomer of human Perforin-2 in its pre-pore conformation. The MACPF and P2 domains are depicted in light green and orange respectively. The carboxy-terminal region is shown in tan. Glycans attached to N185 and N269 are shown as space filling models. Also shown are the positions of deleterious missense (magenta) and nonsense (cyan) mutations (90, 91). Residue numbering is relative to relative to UniProt entry Q2M385 (https://www.uniprot.org/uniprot/Q2M385). **(B)** Killing of intracellular *S*. Typhimurium by neutrophils isolated from the blood of a donor carrying a heterozygous Y430* nonsense mutation or healthy controls. Relative bacterial colony forming units are reported as Log2 Fold Change = log2(CFU at time X) – log2(CFU at time initial). **P < 0.01 by 2way ANOVA. This figure was adapted from previously published work (91) under the CC BY 4.0 license. Molecular graphics were rendered with UCSF ChimeraX from PDB file 6U2J (http://www.rcsb.org/structure/6U2J) (21).

The Genome Aggregation Database (gnomAD, https://gnomad.broadinstitute.org/) catalogs 432 missense (codon changes) and 23 nonsense (premature stop codon) mutations in human *Mpeg1* (89). With few exceptions these mutations are heterozygous and to date only five have been functionally evaluated (90, 91). In one study a young adult female with a history of recurrent polymicrobial skin infections was found to have a heterozygous nonsense mutation in codon Tyr430*; numbering relative to UniProt entry Q2M385 (https://www.uniprot.org/uniprot/Q2M385) (91). This nonsense mutation is within the extended β-hairpin of the P2 domain (**Figure 8A**). It is not known if this truncated protein is stably expressed. But even if it is, it is unlikely to reach endo/phagosomes because it lacks a transmembrane domain and cytosolic tail for retention and intracellular trafficking respectively. Thus, this mutation likely reduces the availability of Perforin-2 within endo/phagosomes. As expected, the ability of the patient’s blood derived phagocytes to eliminate intracellular bacterial pathogens was found to be significantly impaired compared to phagocytes from healthy donors (**Figure 8B**) (91). This impairment is the likely cause of the patient’s clinical presentations and is consistent with the gene dosage effects seen with Perforin-2 heterozygous mice after infection with a variety of pathogens (15, 19).

In another study three *Mpeg1* missense mutations and one nonsense mutation were identified within a cohort of patients with pulmonary nontuberculous mycobacterial infections (90). T73A and P316S are within the MACPF domain while Q398* and P405T reside within the P2 domain (**Figure 8A**). All are heterozygous and each patient presented with a history of pulmonary *Mycobacterium avium* complex as well as nonmycobacterial pulmonary infections. Each of the mutations are apparently deleterious because patient derived cells were less able to kill *M. avium* than cells from age matched controls (90). The researchers subsequently used CRISPR/Cas9 to introduce the mutations into THP-1 cells, a human macrophage-like cell line. As expected, THP-1 cells carrying the missense and nonsense mutations exhibited reduced killing capacity when infected with *M. smegmatis, S.* Typhimurium, and *S. aureus*. Based on their locations within the structures of Perforin-2 T73A and P316S may interfere with the deployment of the pore forming β-strands and pre-pore to pore transition respectively (20, 21). If it is stably expressed Q398* is likely secreted to the extracellular milieu because it lacks the transmembrane domain and cytosolic tail of full length Perforin-2. The impact of P405T is harder to predict as it lies in a more disordered region of the structure (21). However, it is also possible that the observed phenotypes are the result of low protein expression and/or instability since the researchers did not evaluate either (90). In the future higher resolution Perforin-2 structures and greater understanding of the mechanism of acid sensing, pre-pore to pore transition, and pore formation may facilitate testable hypotheses of the clinical impacts of *Mpeg1* missense mutations.

## Bacterial Defenses Against Perforin-2

In resting cells Perforin-2 has a diffuse, perinuclear dispersal. However, it is rapidly relocated to punctate bodies upon exposure to PAMPs or infection (19). Some of these punctate bodies are likely phagosomes because Perforin-2 has been shown to co-localize with phagocytosed bacteria (15). This is consistent with a proteomic study that found Perforin-2 co-resides with subunits of the phagocytic NAPDH oxidase, proton transporters, and many other antimicrobial effectors of phagosomes (17). Other punctate bodies may be sorting endosomes in the process of delivering Perforin-2 to phagosomes because another proteomic study found Perforin-2 in endosomes following phagocytosis of latex beads by macrophages (18). LPS stimulation of bone marrow derived macrophages has also been shown to increase the abundance of Perforin-2 in endo-lysosomes compared to untreated cells (17).

**Figure 9:**
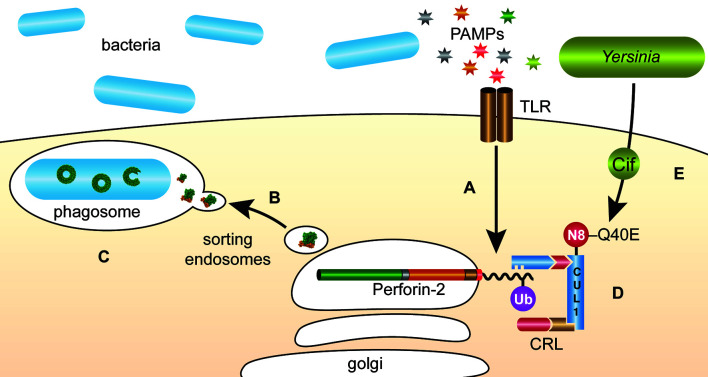
*Y. pseudotuberculosis* and EPEC deploy Cifs to block the delivery of Perforin-2 to phagosomes. (A) The cytosolic tail of Perforin-2 is ubiquitinated in response to PAMPs such as LPS. (B) Ubiquitinated Perforin-2 is rapidly delivered to phagosomes where it oligomerizes and (C) phagosome acidification induces the pre-pore to pore transition. (D) The ligase responsible for conjugating ubiquitin to Perforin-2 is a multi-component cullin-RING E3 ubiquitin ligase (CRL) whose activity is itself dependent upon cullin neddylation. (E) Pathogenic *Y. pseudotuberculosis* and EPEC use Type III secretion systems to inject Cifs into the cytosol of host cells where they deamidate Gln40 of NEDD8. The enzymatic conversion of Gln to Glu inactivates the CRL and thus prevents ubiquitin dependent intracellular trafficking of Perforin-2. N8, NEDD8; Ub, ubiquitin; CUL1, cullin 1.

**Figure 10:**
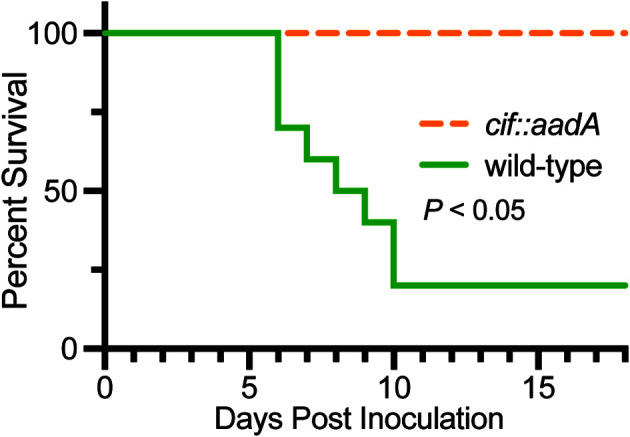
Bacteria lacking the anti-Perforin-2 effector Cif are attenuated *in vivo*. Survival curves of C57BL/6 mice were orogastrically inoculated with 10^8^ CFU wild-type *Y. pseudotuberculosis* or an isogenic *cif* mutant. Significance was calculated by log-rank (Mantel–Cox) test. This figure was adapted from work previously published under the CC BY 4.0 license (19).

The intracellular trafficking of Perforin-2 is driven by PAMP-dependent ubiquitination — most likely monoubiquitination—of one or more conserved lysines in its short cytosolic tail (19). Mutagenesis of the three most conserved lysines abolished the formation of punctate bodies and Perforin-2 dependent killing of bacteria. Ubiquitination of Perforin-2’s cytosolic tail was further shown to be dependent upon a cullin-RING E3 ubiquitin ligase (CRL) complex containing cullin-1 and βTrCP (**Figure 9**) (19). This led to the hypothesis that certain pathogens may deploy Cifs to block the intracellular trafficking of Perforin-2 because CRL activity is dependent upon the ubiquitin like molecule NEDD8 and Cifs are NEDD8 deamidases (92–96). As predicted, the researchers found that wild-type but not *cif* mutants of *Y. pseudotuberculosis* and enteropathogenic *E. coli* (EPEC) blocked the ubiquitination and intracellular trafficking of Perforin-2, as well as Perforin-2 dependent killing (19). In vivo ca. 80% of C57BL/6 mice infected with wild-type *Y. pseudotuberculosis* perished while all mice infected with a *cif* mutant survived (**Figure 10**). This difference was abolished when the two bacterial strains were used to infect *Mpeg1* −/− mice. Although Cif inactivation of CRLs has broad cellular consequences, the latter results suggest that inhibition of Perforin-2 is the primary objective and most consequential effect. While Cifs are just one example of anti-Perforin-2 effectors bacterial pathogens express a multitude of effectors aimed at promoting their survival; many of which have yet to be fully characterized (97). Given that Perforin-2 is a recently described immune effector, it is reasonable to predict that other anti-Perforin-2 effectors will be discovered as the field matures.

## Discussion

Relative to other MACPF proteins of innate immune systems, the field of Perforin-2 research is relatively new even though the gene that encodes Perforin-2, *Mpeg1*, is likely the most ancient of the MACPF encoding genes (14). Nevertheless in recent years vertebrate studies have established that Perforin-2 has broad spectrum bactericidal activity that significantly limits pathogen proliferation and dissemination *in vivo* (15, 19, 26, 52–54, 57, 72, 90). There is also evidence to suggest that Perforin-2 functions similarly in invertebrates; although, such studies are hampered by the lack of animal and tractable cell culture models (27, 28, 30, 31, 33, 38, 39). The recent structural determinations of both mouse and human Perforin-2 polymers have further provided significant insight with regards to the mechanism(s) of pore formation (20, 21). Eventually such high-resolution structures may afford greater understanding of the clinical impacts of *Mpeg1* missense mutations amongst the human population.

*In vitro* recombinant mammalian Perforin-2 spontaneously polymerizes and one of the most significant advancements from *in vitro* studies was the discovery that the pre-pore to pore transition is acid dependent (20, 21). In retrospect the fact that low pH drives pore formation seems intuitive given that Perforin-2 is deployed to acidic phagosomes. Acid dependency may also be a safety mechanism, ensuring that Perforin-2 remains in a latent state so as not to damage the vesicular and cellular membranes of the phagocyte. Acid dependency likely evolved very early as lower metazoans such as sponges, corals, and mollusks have been shown to express *Mpeg1* and have macrophage-like cells that phagocytose and eliminate foreign invaders through the use of lysosomal enzymes, reactive oxygen species, and cellular acidification (98–103). However, the exact mechanism of acid driven pore formation remains to be elucidated. One possibility is that acidification removes inhibitory inter- or intra-domain contacts that prevent pore formation. Clearly one of the most important objectives in this area will be to discover the acid-dependent trigger within Perforin-2.

It is also not yet known if Perforin-2 can breach bacterial membranes by itself or if it requires the assistance of other host proteins. Our review and evaluation of the *in vitro* systems reported to date raises significant doubts that Perforin-2 dependent killing was actually observed with recombinant protein (27, 29–31, 33, 34, 38, 39, 43). Our first major concern is that some researchers opted to purify the MACPF domain in the absence of the P2 domain (29, 31, 43). However, we now know that the P2 domain forms the outer ring of Perforin-2 polymers with extensive surface area packed between the two domains and neighboring subunits (**Figure 3**) (20, 21). Thus, it seems improbable that the MACPF domain of Perforin-2 would polymerize in the absence of the P2 domain. In addition, there is evidence that the extended β-hairpin of the P2 domain mediates the initial interactions with target membranes (**Figure 2**) (20). Therefore, even if the MACPF domain does polymerize it is unclear how it would target bacterial membranes. We also note that all studies with recombinant Perforin-2 did not convincingly demonstrate bacterial killing (27, 29, 31, 34, 43). Rather, most simply reported changes in the optical absorbance of bacterial cultures. As discussed below there is also the possibility that glycosylation is essential for Perforin-2 folding, polymerization, or pore formation/stabilization. These post-translational modifications would be absent in the studies that produced recombinant Perforin-2 or its MACPF in *E. coli* (27, 29, 31, 34, 43). Although claims of recombinant Perforin-2’s bactericidal activity are to date unconvincing, the development of a technically sound *in vitro* bactericidal assay would be a substantial advance that would facilitate further mechanistic investigations.

Among other outstanding questions is the necessity of post-translational modifications; specifically, glycosylation. Two independent structural studies observed glycosylation of Asn185 and Asn269 (20, 21). These residues are conserved and glycosylated in both human and mouse Perforin-2. Despite their conservation it is not known if the glycans are required for protein stability/folding, oligomerization, or pore formation/stabilization. Elucidating the functional and mechanistic consequences of Perforin-2 glycosylation may have clinical implications because missense mutations of both Asn185 and Ans269 are present within the human population (gnomAD, https://gnomad.broadinstitute.org/).

There are also significant gaps in our understanding of the intracellular trafficking of Perforin-2. Although it has been shown that ubiquitination of Perforin-2’s cytosolic tail drives trafficking (19), the mechanism(s) of intracellular trafficking and delivery to the phagosome is largely unknown. Likewise, the linkage between PAMP/TLR signaling and ubiquitination is unclear. This may involve the activation of a kinase that phosphorylates the cytosolic tail of Perforin-2 prior to CRL-dependent ubiquitination. However, this upstream pathway remains in the realm of the hypothetical.

Perforin-2 may also require proteolytic processing to release it from its transmembrane domain and facilitate subsequent polymerization. Indeed Perforin-2 fragments have been observed after cellular infection (15). Perhaps the cleavage products are relevant to polymerization and pore formation. Alternatively, they may be mechanistically insignificant degradation products. Although it is not yet known which of these two scenarios is correct, we and others suspect that tethering to a vacuolar membrane is inhibitory; preventing polymerization (20, 104, 105). In our “safety tether” hypothesis Perforin-2 is maintained as a monomer as long as it exists as a Type I transmembrane protein. However we also note that there is evidence for Perforin-2 dependent autolysis under certain circumstances. For example, Eckhard Podack used negative stain transmission electron microscopy to image apparent Perforin-2 polymers with membrane preparations of HEK-293 cells engineered to overexpress Perforin-2-GFP (15). Of potential relevance to our safety tether hypothesis, Perforin-2 polymers were only observed after partial trypsin digest. Polymers were not observed with undigested preparations. More recently Hung et al. have shown that Perforin-2 is required for the release of IL-33 from dendritic cells (106). Because IL-33 lacks a signal peptide, the authors propose that Perforin-2 forms pores in the plasma membrane to release cytosolic IL-33. Although the supporting data is quite convincing, the study raises many mechanistic questions. For example, does Perforin-2 attack the plasma membrane from the exterior or interior of the cell? In either case does pore formation involve proteolytic cleavage and/or low pH? In addition there is no known mechanism for the selective gating of Perforin-2 pores. Therefore, it seems likely that IL-33 secreting dendritic cells would also release a plethora of cytosolic molecules. Is Perforin-2 autolysis ultimately lethal or is autolysis somehow mitigated to prevent lethality? Addressing these and other questions will provide greater understanding of Perforin-2 functionality and may also identify novel clinical avenues that could be used to treat diseases associated with Perforin-2 deficiencies.

Although Perforin-2 research has made considerable progress from the perspective of the host, less is known about pathogen strategies to survive Perforin-2. To date, Cifs are the only class of anti-Perforin-2 effectors to be discovered. Given the persistence of Perforin-2 throughout diverse organisms, it is reasonable to expect that many other pathogenic countermeasures remain undiscovered. With sufficient interest progress in this area is possible and particularly suited to unbiased screens such as Tn-Seq. Once a candidate anti-Perforin-2 effector is identified, it can be thought of as a molecular probe that can elucidate the cellular and molecular mechanisms of Perforin-2 dependent killing. Of course, more passive survival strategies — such as alteration of the bacterial envelope or cell wall — are also possible. Despite numerous unknowns, it is clear that this field is poised for expansion and discovery.

## Author Contributions

LM, ZR, and GM developed the concept, critically reviewed the literature, and wrote various sections of the manuscript. GM was responsible for figure design and layout. All authors participated in the manuscript revision. All authors contributed to the article and approved the submitted version.

## Funding

Perforin-2 research in the laboratory of GM is supported by the National Institute of Allergy and Infectious Diseases of the National Institutes of Health under award numbers R01AI110810 and R21AI156567. The content of this publication is solely the responsibility of the authors and does not necessarily represent the official views of the National Institutes of Health.

## Conflict of Interest

The authors declare that the research was conducted in the absence of any commercial or financial relationships that could be construed as a potential conflict of interest.
